# Well-Child Visits for Early Detection and Management of Maternal Postpartum Hypertensive Disorders

**DOI:** 10.1001/jamanetworkopen.2024.16844

**Published:** 2024-06-13

**Authors:** Farah H. Amro, Kim C. Smith, Syed S. Hashmi, Michelle S. Barratt, Rachel Carlson, Kristen Mariah Sankey, Michal Fishel Bartal, Sean C. Blackwell, Suneet P. Chauhan, Baha M. Sibai

**Affiliations:** 1 Division of Maternal-Fetal Medicine, Department of Obstetrics, Gynecology, and Reproductive Sciences, McGovern Medical School, The University of Texas Health Science Center at Houston; 2 Division of Community & General Pediatrics, Department of Pediatrics, McGovern Medical School, The University of Texas Health Science Center at Houston; 3 Department of Obstetrics, Gynecology, and Reproductive Sciences, McGovern Medical School, The University of Texas Health Science Center at Houston; 4 Department of Maternal-Fetal Medicine, Delaware Center of Maternal-Fetal Medicine, Newark, Deleware

## Abstract

**Importance:**

Innovative approaches are needed to address the increasing rate of postpartum morbidity and mortality associated with hypertensive disorders.

**Objective:**

To determine whether assessing maternal blood pressure (BP) and associated symptoms at time of well-child visits is associated with increased detection of postpartum preeclampsia and need for hospitalization for medical management.

**Design, Setting, and Participants:**

This is a pre-post quality improvement (QI) study. Individuals who attended the well-child visits between preimplementation (December 2017 to December 2018) were compared with individuals who enrolled after the implementation of the QI program (March 2019 to December 2019). Individuals were enrolled at an academic pediatric clinic. Eligible participants included birth mothers who delivered at the hospital and brought their newborn for well-child check at 2 days, 2 weeks, and 2 months. A total of 620 individuals were screened in the preintervention cohort and 680 individuals were screened in the QI program. Data was analyzed from March to July 2022.

**Exposures:**

BP evaluation and preeclampsia symptoms screening were performed at the time of the well-child visit. A management algorithm—with criteria for routine or early postpartum visits, or prompt referral to the obstetric emergency department—was followed.

**Main Outcome and Measures:**

Readmission due to postpartum preeclampsia. Comparisons across groups were performed using a Fisher exact test for categorical variables, and *t* tests or Mann-Whitney tests for continuous variables.

**Results:**

A total of 595 individuals (mean [SD] age, 27.2 [6.1] years) were eligible for analysis in the preintervention cohort and 565 individuals (mean [SD] age, 27.0 [5.8] years) were eligible in the postintervention cohort. Baseline demographic information including age, race and ethnicity, body mass index, nulliparity, and factors associated with increased risk for preeclampsia were not significantly different in the preintervention cohort and postintervention QI program. The rate of readmission for postpartum preeclampsia differed significantly in the preintervention cohort (13 individuals [2.1%]) and the postintervention cohort (29 individuals [5.6%]) (*P* = .007). In the postintervention QI cohort, there was a significantly earlier time frame of readmission (median [IQR] 10.0 [10.0-11.0] days post partum for preintervention vs 7.0 [6.0-10.5] days post partum for postintervention; *P* = .001). In both time periods, a total of 42 patients were readmitted due to postpartum preeclampsia, of which 21 (50%) had de novo postpartum preeclampsia.

**Conclusions and Relevance:**

This QI program allowed for increased and earlier readmission due to postpartum preeclampsia. Further studies confirming generalizability and mitigating associated adverse outcomes are needed.

## Key Points

**Question:**

Does maternal evaluation during well-child visits offer an opportunity for detection of postpartum hypertensive disorders?

**Findings:**

In this quality improvement study of 595 individuals preintervention and 565 individuals postintervention, there was a statistically significant increase in readmission rates due to detection of maternal postpartum hypertensive disorders from preintervention (2%) to postintervention (6%). Readmission also occurred earlier for those in the postintervention group.

**Meaning:**

The QI program allowed for increased and earlier detection of maternal postpartum hypertensive disorders that required readmission, suggesting that this approach may have merit; however, further studies are still needed to confirm generalizability.

## Teaser

This quality improvement study analyzes whether assessing maternal blood pressure and associated symptoms at the time of well-child visits vs traditional postpartum visits is associated with increased detection of postpartum preeclampsia.

## Introduction

In the US, every 10 days, 1 maternal death occurs due to hypertensive disorders of pregnancy (HDP),^1^ with 40% occurring within 6 weeks post partum.^2^ Postpartum hypertension—be it HDP or de novo hypertension after childbirth^3^—is also a leading cause of severe maternal morbidity. Between 1998 and 2009, eclampsia has increased by 64% (from 1.76 to 2.90 per 10 000 delivery hospitalizations), acute myocardial infarction by 131% (from 0.18 to 0.42 per 10 000 delivery hospitalizations), and cerebrovascular disorders by 107% (from 1.51 to 3.13 per 10 000 delivery hospitalizations).^4^ There is, however, an acknowledged paucity of data on hypertensive disorders in the postpartum period,^3,5,6,7^ specifically on de novo postpartum hypertension. The reasons for insufficient data on postpartum hypertension are multifactorial. First, in clinical practice, in the absence of HDP, individuals are not usually seen for their postpartum visit until 2 weeks after a cesarean delivery and 4 to 6 weeks after a vaginal delivery, and therefore will not have an earlier postpartum blood pressure (BP) recorded.^3^ Second, individuals may not keep their postpartum appointments due to several stressors such as lack of sleep, fatigue, breastfeeding difficulties, and adjustment to the newborn.^8^ Third, if an individual presents for an acute BP evaluation, it is done in the emergency department setting by the nonobstetric clinician whereby the diagnosis of hypertensive disorders of pregnancy may be missed.^9^

Recognizing the underutilization of postpartum care and the high morbidity and mortality during the postpartum period, the American College of Obstetrics and Gynecology (ACOG) recently updated their fourth trimester guidelines.^8^ With recommendations for earlier and more frequent visits in the new paradigm shift, ACOG recommends that those with a high risk hypertensive status be evaluated 3 to 10 days after delivery for a BP check.^8^ However, this recommendation has been challenging to adopt in clinical practice.^8^ A recent randomized clinical trial from our institution^10^ demonstrated that only 50% of patients at high risk attended their 7- to 10- day postpartum visit. Furthermore, even if these new fourth trimester guidelines were adhered to, it would not allow for timely detection of de novo postpartum preeclampsia (ie, in patients that are otherwise deemed low risk), which have a reported prevalence of 0.3% to 27.5%.^3^

In comparison, neonatal well-child visits are routinely attended more frequently (approximately 90%) than postpartum visits and as early as 2 days after hospital discharge for all newborns.^11^ Thus, these visits present an opportunity for evaluation of maternal BP and symptoms of HDP.^8,9^

To capitalize on earlier and more frequent health care encounters afforded by the well-child visits compared with postpartum visits, we developed a quality improvement (QI) program to assess maternal BP and symptoms of HDP at the time of well-child visits in the first 2 months post partum. The goal of this study was to assess the feasibility of capturing maternal BP data at these pediatric visits and to compare the prevalence of HDP captured by this program vs current standard of care methods.

## Methods

### Study Design and Setting

This QI study was approved by the institutional review board of the University of Texas Health Science Center at Houston with a waiver of informed consent because this was a minimal risk study. The study adhered to the revised Standards for Quality Improvement Reporting Excellence (SQUIRE 2.0) reporting guidelines. Our preintervention and postintervention QI program was conducted in a pediatrics clinic at an academic medical center that is staffed by board-certified pediatricians who oversee pediatric resident trainees. This is a low-risk pediatrics clinic that does not evaluate premature infants (delivery before 37 weeks) with pulmonary issues. After obtaining the institutional review board approval from our institution, the QI program commenced in March 2019.

The preintervention cohort consisted of biological mothers that accompanied their newborns to our pediatric clinic between December 2017 and December 2018, prior to the initiation of our QI program. This data on the preintervention cohort was abstracted retrospectively utilizing the electronic medical record system. We identified whether the biological mother accompanied the newborn based on their identification information being scanned into the newborn’s medical record within the 2-month time frame after delivery. The postintervention (QI) cohort was prospectively enrolled and consisted of biological mothers accompanying their newborns to the same clinic for the next 10 consecutive months (March to December 2019). These individuals were approached by the pediatrics clinician about enrollment in our QI program at the time of routine well-child visits (2 days after discharge, 2 weeks, and 2 months).

### Selection of Participants

**Figure 1.**
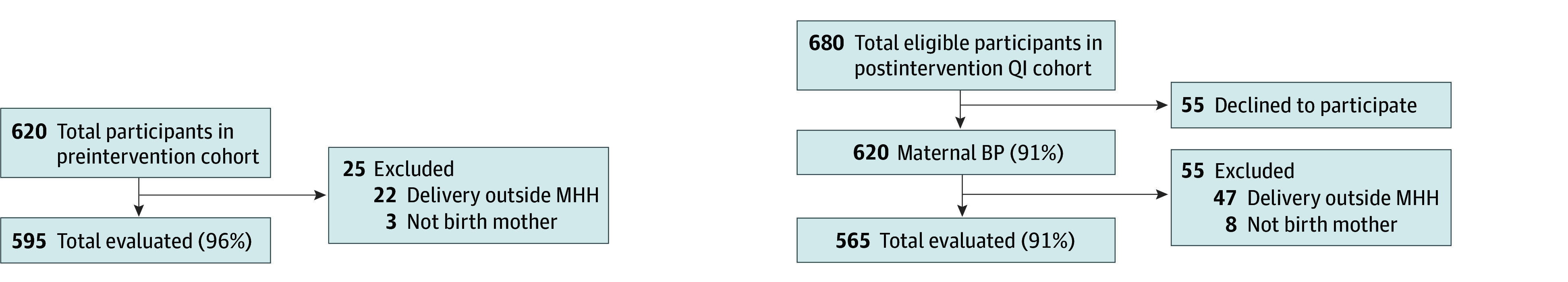
Screening and Enrollment Flow Chart BP indicates blood pressure; MHH indicates Memorial Hermann Hospital; QI, quality improvement.

Both in the preintervention and postintervention cohorts, individuals were included if they delivered at our tertiary care academic medical center and their newborns were evaluated in our pediatric clinics. In the postintervention cohort, all individuals who presented to the pediatrics clinic with their newborns were approached for verbal consent. They were excluded if they had delivered at an outside facility (no access to medical records), or if they were not the birth mothers (Figure 1).

### Interventions

**Figure 2.**
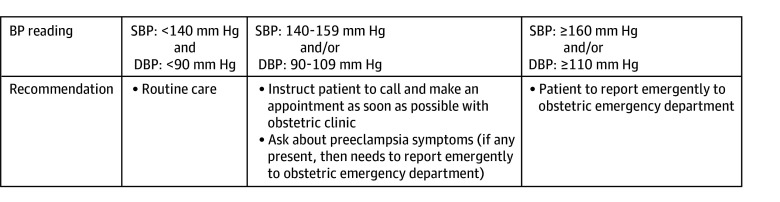
Management Algorithm BP indicates blood presure; DBP indicates diastolic blood pressure; SBP, systolic blood pressure.

During the visit, a trained medical assistant obtained the BP using a semiautomated BP cuff according to accepted BP detection criteria (per American College of Cardiology and American Hospital Association guidelines).^12^ Mothers were instructed to sit down for their BP evaluation, and if an elevated recording was obtained (defined as systolic BP [SBP] ≥140 mm Hg and/or diastolic BP [DBP] ≥90 mm Hg), then the BP measurement was repeated after 5 minutes. The second BP recording would be used for algorithm recommendations. The medical assistant also evaluated for preeclampsia symptoms (headache, scotomata, right upper quadrant pain, or shortness of breath). A management algorithm was then followed (Figure 2). Individuals were instructed to go to the obstetrics emergency department (OB-ED) for evaluation if they had a mild-range BP (defined as SBP ≥140 mm Hg and/or DBP ≥90 mm Hg) with symptoms of preeclampsia, or a severe-range BP (defined as SBP ≥160 mm Hg and/or DBP ≥110 mm Hg) as described in Figure 2.

**Figure 3.**
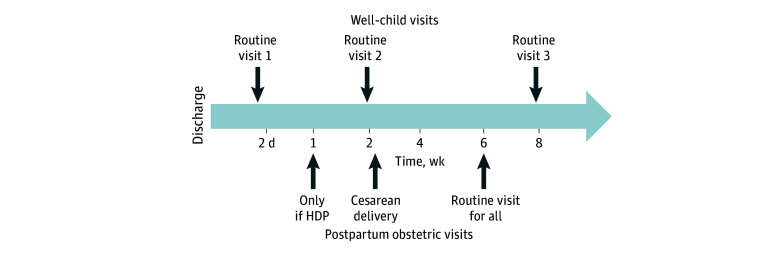
Timing of Well-Child Visits and Postpartum Obstetric Visits Overview HDP indicates hypertensive disorder of pregnancy.

Both cohorts still adhered to our standard obstetric postpartum follow-up visit schedule, which includes a 7- to 10-day postpartum follow-up BP evaluation for patients with HDP as recommended by ACOG guidelines, 2-week follow-up for cesarean delivery, and 4 to 6 weeks for all mothers at our obstetric clinic. The evaluation at the well-child visits was in addition to our routine obstetric postpartum follow-up recommendations (Figure 3).

### Measurements

BPs at time of presentation to the newborn visit (as well as during the hospital admission), maternal symptoms, demographics, and delivery and readmission information were obtained through detailed electronic medical record abstractions using REDCap. Race and ethnicity were identified based on patient report in the electronic health records. Reported race and ethnicity categories included African American, Asian, Hispanic, White, and other (defined as any other race or ethnicity not otherwise specified). Race and ethnicity were included to account for health disparities.

### Outcomes

The primary outcome was postpartum readmission due to preeclampsia with severe features within 2 months after delivery.^13^ We utilized the ACOG definitions of HDP. Preeclampsia without severe features was defined as new-onset hypertension (SBP ≥140 mm Hg and/or DBP ≥90 mm Hg on 2 occasions at least 4 hours apart from 20 weeks of gestation until the postpartum period) and proteinuria (spot urine and protein creatinine ratio ≥0.3 mg/dL [to convert to micromoles per liter, multiply by 88.4]). Gestational hypertension was defined using the same BP criteria as preeclampsia without severe features but with the absence of proteinuria. A diagnosis of preeclampsia with severe features was made in the setting of either new-onset hypertension as defined previously or severe hypertension (sustained SBP ≥160 mm Hg and/or DBP ≥110 mm Hg) and new-onset of any of the following features: thrombocytopenia (less than 100 × 10^3^/µL [to convert to × 109/L, multiply by 1]), kidney insufficiency (serum creatinine concentrations greater than 1.1 mg/dL or a doubling of serum creatinine concentration in the absence of other kidney disease), impaired liver function (as evidenced by elevation of liver transaminases ≥twice the upper limit of normal), pulmonary edema, or cerebral or visual symptoms. A diagnosis of superimposed preeclampsia was made in the setting of chronic hypertension (hypertension before 20 weeks of gestation) plus any of the aforementioned features (with or without severe hypertension).^13^ De novo postpartum preeclampsia was defined as developing preeclampsia with severe features in the postpartum period after discharge from delivery admission with the absence of chronic hypertension or an underlying HDP (gestational hypertension, preeclampsia with or without severe features, or superimposed preeclampsia).^3^ The secondary outcomes included latency from discharge to readmission, preeclampsia symptoms at time of readmission, BP at time of presentation for readmission, and serious morbidity (defined as pulmonary edema, stroke, or reversible cerebral vasoconstriction syndrome).

### Statistical Analysis

Categorical variables were described as frequencies with percentages. Continuous variables were described as means with SDs for normally distributed data or medians with IQRs for data that was not normally distributed. Comparisons across groups were performed using a Fisher exact test for categorical variables, and *t* tests or Mann-Whitney tests for continuous variables. Statistical significance was assumed at a type 1 error rate of 5%. All analyses were performed using Stata version 14 (StataCorp). Statistical analysis took place from March to July 2022.

## Results

In the preintervention cohort, a total of 620 individuals were screened, of which 25 were excluded, leaving 595 individuals (96%; mean [SD] age, 27.2 [6.1] years) for analysis. In the postintervention cohort, a total of 680 individuals were approached in the pediatrics clinic for verbal consent to enroll in our QI program, of which 620 (91%) agreed to participate. Fifty-five were not eligible for analysis (47 due to delivery outside of our hospital system and 8 were not the birth mother). Our analysis focused on 565 individuals (91%; mean [SD] age, 27.0 [5.8] years) who met the inclusion criteria (Figure 3).

Maternal demographics were not significantly different between the 2 groups; this included age, race or ethnicity, nulliparity, and BMI (Table 1). Of the entire 2 cohorts combined, more than two-thirds were from racial and ethnic minority groups (889 individuals [77%]) and almost 80% had public insurance (913 individuals [79%]). Additionally, factors associated with increased risk for preeclampsia such as history of pregestational diabetes, chronic hypertension, preeclampsia in prior pregnancy, and HDP diagnosis in current pregnancy were also not significantly different between the 2 groups.

**Table 1.** Maternal Baseline Characteristics

Characteristic	Participants, No. (%)		*P* value
	Preintervention (N = 595)	Postintervention quality improvement (N = 565)	
Age, mean (SD), y	27.2 (6.1)	27.0 (5.8)	.57
Race or ethnicity			
African American	295 (50)	302 (54)	.29
Asian	13 (2)	11 (2)	
Hispanic	154 (23)	138 (2)	
White	56 (9)	61 (11)	
Other^a^	77 (13)	53 (9)	
Nulliparity	173 (29)	171 (30)	.65
Multifetal gestation	18 (3)	14 (3)	.60
Cesarean delivery	215 (35)	201 (36)	.67
Body mass index at delivery, median (IQR)^b^	32.1 (27.7-37.0)	32.5 (28.2-38.0)	.14
Body mass index >30^b^	363 (63)	357 (64)	.62
Pregestational diabetes	49 (8)	48 (9)	.78
Gestational diabetes	27 (5)	30 (5.)	.40
Chronic hypertension	56 (9)	56 (10)	.77
History of preeclampsia in prior pregnancy	42 (10)	42 (11)	.82
Hypertensive disorder of pregnancy diagnosis in current pregnancy^c^	138 (23)	143 (25)	.41

^a^ Other was defined as participants who identified as any other race or ethnicity not otherwise specified.

^b^ Body mass index was calculated as weight in kilograms divided by height in meters squared.

^c^ Received a diagnosis antepartum, intrapartum, or postpartum following delivery admission. Hypertensive disorder of pregnancy included gestational hypertension with or without severe features, preeclampsia with or without severe features, and eclampsia.

In both time periods, a total of 42 patients were readmitted due to postpartum preeclampsia, of which 21 (50%) had de novo postpartum preeclampsia. A total of 33 OB-ED visits were recommended in the QI program using the management algorithm, and they resulted in postpartum preeclampsia readmission of 29 individuals (88%). The rate of readmission due to postpartum preeclampsia was significantly higher in the QI postintervention cohort compared to the preintervention cohort (29 of 565 individuals [2%] vs 13 of 595 individuals [6%]; *P* = .007). When comparing baseline demographics in those readmitted before and after implementation of the program, race and ethnicity in the 2 groups were significantly different in that individuals in the postintervention group were more likely to be African American than the preintervention group (4 of 13 individuals [31%] vs 26 of 29 individuals [90%]; *P* < .001). HDP diagnosis made prior to postpartum readmission was noted to be less frequent in the preintervention group (3 of 13 individuals [23%] vs 18 of 29 individuals [62%]; *P* = .04) (eTable in Supplement 1). Of the 29 patients in the postintervention QI program with preeclampsia, 12 (41%) were asymptomatic.

Table 2 compares the presentations of those readmitted due to postpartum preeclampsia and outcomes. The primary reason for presentation to the OB-ED was not related to preeclampsia in 3 of 13 individuals in the preintervention cohort (23%) and 2 of 29 individuals in the postintervention QI program (7%). Of those readmitted, those in the preintervention cohort were more likely to be symptomatic, although the finding was not statistically significant (9 of 13 individuals [69%] vs 17 of 29 individuals [59%]; *P* = .75); with the most common symptom at time of presentation being headache. Serious morbidity occurred in both groups, including 1 case of pulmonary edema in the preintervention cohort and 2 cases of pulmonary edema and 1 case of reversible cerebral vasoconstriction syndrome in the postintervention cohort.

**Table 2.** Postpartum Preeclampsia Readmission Presentation and Outcomes

Characteristic	Participants, No. (%)		*P* value
	Preintervention (n = 13)	Postintervention quality improvement (n = 29)	
Systolic blood pressure at OB-ED on presentation, median (IQR), mm Hg	170.0 (160.0-179.0)	162.0 (148.0-172.0)	.19
Diastolic blood pressure at OB-ED on presentation, median (IQR), mm Hg	90.0 (78.0-96.0)	94.0 (90.0-97.0)	.28
Required antihypertensive intravenous therapy	6 (46)	17 (59)	.68
Started oral antihypertensive medications	8 (62)	24 (83)	.57
Increased titrated medications or changed oral antihypertensive medications	2 (15)	3 (10)	.68
Asymptomatic	4 (31)	12 (41)	.25
Symptoms	9 (69)	17 (59)	
Headache	6 (46)	16 (55)	.75
Shortness of breath	2 (15)	2 (7)	
Scotomata	1 (8)	0	
Acute right upper quadrant pain	1 (8)	1 (3)	
Laboratory abnormality	2 (15)	6 (21)	
Creatinine	2 (15)	5 (17)	.74
Liver enzymes	0	1 (3)	
Platelets	0	0	
Presentation due to other reason at OB-ED	3 (23)	2 (7)	.20
De novo postpartum preeclampsia^a^	10 (77)	11 (38)	<.001
Postpartum day of readmission, median (IQR)	10.0 (10.0-11.0)	7.0 (6.0-10.5)	<.001
Latency from discharge to readmission, median (IQR)	8.0 (8.0-11.0)	5.0 (2.5-7.0)	.18
Serious morbidity	1 (8)^b^	3 (10)^c^	.79
Duration of stay during readmission, median (IQR) d	1.5 (1.0-2.0)	2.0 (1.0-2.0)	.81

Abbreviation: OB-ED, obstetric emergency department.

^a^ Did not have gestational hypertension, preeclampsia, or superimposed preeclampsia prior to discharge from delivery admission.

^b^ Pulmonary edema.

^c^ Pulmonary edema (2 participants) and reversible cerebral vasoconstriction syndrome (1 participant).

On presentation to the OB-ED, the BP parameters were not significantly different between the 2 groups. Intravenous antihypertensives were required for 6 of 13 individuals in the preintervention group (46%) in comparison with 17 of 29 individuals in the postintervention group (59%). Oral antihypertensives were initiated for 8 of 13 individuals in the preintervention cohort (62%) and 24 of 29 individuals in the postintervention cohort (83%). The diagnosis of de novo postpartum preeclampsia was more likely to be present in the preintervention cohort (10 of 13 individuals [77%] vs 11 of 29 individuals [38%]; *P* < .001). The day of readmission differed significantly in the 2 groups; in the preintervention cohort, the median (IQR) day of readmission was postpartum day 10.0 (10.0-11.0) for the preintervention cohort vs day 7.0 (6.0-10.5) in the postintervention cohort (*P* < .001). The duration of stay during readmission between both groups was similar (Table 2).

## Discussion

There are 3 key findings of this QI study. First, our study suggests that capturing information on maternal blood pressure and associated symptoms during well-child visits in the first 2 postnatal months is a practicable option for postnatal screening of HDP. More than 95% of the children were accompanied by their birth mothers during the well-child visits. Second, the improved screening for HDP in the postintervention group compared with the preintervention group resulted in diagnosing HDP in cases where it would have potentially been missed if screening was left to postnatal maternal clinic visits. This was evident by the significantly higher number of mothers being referred to the OB-ED and being admitted due to postpartum preeclampsia. Third, the improvement in diagnosis also resulted in better clinical outcomes with respect to improved recognition and diagnosis of de novo HDP and an earlier readmission for HDP in the postintervention group compared with the preintervention group.

Additionally, we observed that individuals readmitted for postpartum preeclampsia were more likely to be African American and to have an underlying HDP diagnosis prior to delivery admission discharge. This finding mirrors previous studies^14,15^ that have also shown an increased risk of readmission post partum due to preeclampsia in African American individuals, with reports of non-Hispanic African American individuals being 80% more likely to be readmitted in comparison to non-Hispanic White individuals. Disparities in medical care may have contributed to the different racial distribution in the preintervention and postintervention cohorts, suggesting that implicit bias may have impacted clinical decision making and behavior during encounters with African American patients, resulting in lower rates of readmission.^16,17^ With the QI program, the BP evaluation was done by another physician and referral to the OB-ED by that physician may have mitigated those biases.

Another finding in our postintervention cohort, which is echoed in other studies, is that known HDP from delivery admission is associated with increased risk for postpartum preeclampsia readmission.^18,19^ However, as many as 50% of those readmitted would be considered a de novo diagnosis, which is similar to what is reported by Matthys et al^17^ (66%) and Al-Safi et al^18^ (63.2%). Current ACOG guidelines do not address the need for early follow-up for those that do not have a known hypertension diagnosis and are considered low risk by ACOG.^8^ Thus, these patients would otherwise have not been identified. While it would be challenging to recommend early follow-up to all individuals who deliver due to the increased burden to the health care system, the presence of mandatory well-child visits for all children at early intervals will help address this in a cost-effective approach.

Similar to previous studies,^20,21^ we also found that as many as 41% of our postintervention cohort patients were asymptomatic on presentation for readmission due to preeclampsia. Of those that were symptomatic the most common symptom was headache.

The postpartum period presents a critical period for the health of a mother and their infant with increased maternal^2^ and neonatal morbidity and mortality in this time frame.^22^ Thus, it is imperative to seek medical care, however the postpartum period is unique in its additional barriers. While a mother is recovering from childbirth and adjusting to the newborn, there are several added fourth trimester stressors such as lack of sleep, fatigue, pain, and breastfeeding difficulties to name a few.^8^ Therefore, seeking fragmented care between the maternal and pediatric health care clinicians can present an added challenge, and at these times, as evidenced by our obstetric postpartum visit attendance rates, attending the maternal care clinician visits tends to take a back seat.^15^

To help address this issue, new strategies are being proposed to improve detection of postpartum hypertension, including the use of remote BP cuff monitoring systems.^14,23^ However, these strategies rely on the patient measuring and reporting their own BP, which is subject to patient compliance and has the potential for inaccuracies.^24^ Furthermore, hypertensive disorders occurred de novo post partum in 50% of our participants and they would not be targeted for evaluation by these strategies, which are implemented for patients at high risk.^8^

Our approach of utilizing the well-child visits allows for a more comprehensive care of the mother-infant dyad with a multidisciplinary approach because the health and well-being of the mother and infant are intertwined.^14,15^ The strengths of our study are that it represents a diverse patient population with more than two-thirds of the cohorts from racial and ethnic minority groups and more than 80% having public insurance. Our QI program data was prospectively collected at a tertiary academic center. It also represents a large cohort of patients that were evaluated. It is a novel program and presents a unique opportunity for multidisciplinary involvement in the care of the mother post partum.

### Limitations

A limitation of our study is that because this was a single site study, we did not have access to readmission data from outside hospital systems. It is possible that some of the mothers sought help at other health care facilities and were potentially misclassified with respect to the outcome (postpartum readmission) in our analyses. However, the probability of this misclassification is most likely equal in both cohorts (nondifferential misclassification) because there were no major changes in our institutional policies, geographic catchment area, or sample population demographics between the preintervention and postintervention QI cohorts. Nondifferential outcome misclassifications, especially at the sample sizes present in our study (relatively large sample size that was nearly equally distributed between the 2 groups), are more likely to dampen the effect size without crossing over the null (ie, bias toward the null)^25^ Therefore, any bias introduced by this misclassification is more likely to have resulted in an underestimation of our effect size (differences between groups; ie, the true effect size is likely to be larger than what we report here). Additionally, although a single-site study raises questions about external validity, as a large tertiary–care center study with a large and diverse (across various factors) study sample, we feel that these results are potentially robust and generalizable. Nonetheless, replication of these results is warranted. Additionally, white coat syndrome has also been described in the literature and this may have had an impact on our BP measurement.^26^ However, of the 33 patients sent to the ED for evaluation, 29 (88%) were readmitted. This suggests a high degree of sensitivity (true positives) and, thus, reliability of the evaluations performed at the pediatric well-child visits.

## Conclusions

Maternal evaluation at the time of well-child visits presents a novel opportunity for maternal BP check and preeclampsia symptoms assessment. Our QI study found an increase in the rate of readmission due to postpartum preeclampsia at an earlier time frame with this intervention. Further research is needed to verify and expand the applicability of this approach.

## Notes

Supplement 1. **eTable.** Postpartum Preeclampsia Readmission Baseline Characteristics

Supplement 2.
Data Sharing Statement
