# Supplementary material for: Well-Child Visits for Early Detection and Management of Maternal Postpartum Hypertensive Disorders
Source: JAMA Netw Open. 2024 Jun 13;7(6):e2416844. doi: 10.1001/jamanetworkopen.2024.16844 (PMC11177164; doi:10.1001/jamanetworkopen.2024.16844)
Supplement: Supplement 1. — eTable. Postpartum Preeclampsia Readmission Baseline Characteristics [file jamanetwopen-e2416844-s001.pdf]

Supplemental Online Content

Amro FH, Smith KC, Hashmi SS, et al. Well-child visits for early detection and management of maternal postpartum hypertensive disorders. *JAMA Netw Open*. 2024;7(6):e2416844. doi:10.1001/jamanetworkopen.2024.16844

eTable. Postpartum Preeclampsia Readmission Baseline Characteristics

This supplemental material has been provided by the authors to give readers additional information about their work.

© 2024 Amro FH et al. *JAMA Network Open.*

eTable. Postpartum Preeclampsia Readmission Baseline Characteristics

Characteristic	Pre-intervention Cohort	Post-intervention QI Cohort	P-value
	N= 13	N=29	
Age- year, mean (SD)	29.2 (5.8)	30.3 (5.8)	0.55
Race or Ethnic Group			
African American	4 (30.8)	26 (89.7)	<0.001
Asian	0 (0)	0 (0)	
Hispanic	3 (23.1)	1 (3.5)	
White	1 (7.7)	1 (3.5)	
Other [a]	5 (38.5)	1 (3.5)	
Nulliparity	2 (15)	6 (21)	0.68
Multifetal Gestation	2 (15)	0 (0)	0.09
BMI at delivery, median (IQR)	32 (27 – 38)	38 (32-47)	0.09
BMI > 30 (%)	8 (62)	23 (79)	0.27
CHTN	3 (23)	12 (41)	0.34
Prior pregnancy complicated by preeclampsia	1 (9)	6 (21)	0.65
HDP diagnosis in current pregnancy prior to discharge from delivery admission [b]	3 (23)	18 (62)	0.043
Discharge from delivery admission on BP medication	2 (15)	5 (17)	0.73

SBP= systolic blood pressure, DBP= diastolic blood pressure

[a] Other = participants who identified as “Other” and not to the other 4 race/ethnic groups

[b] antepartum, intrapartum, postpartum in delivery admission

© 2024 Amro FH et al. *JAMA Network Open.*
