# Supplementary material for: Well-Child Visits for Early Detection and Management of Maternal Postpartum Hypertensive Disorders
Source: JAMA Netw Open. 2024 Jun 13;7(6):e2416844. doi: 10.1001/jamanetworkopen.2024.16844 (PMC11177164; doi:10.1001/jamanetworkopen.2024.16844)
Supplement: Supplement 2. — Data Sharing Statement [file jamanetwopen-e2416844-s002.pdf]

Data Sharing Statement

Amro. Well-Child Visits for Early Detection and Management of Maternal Postpartum Hypertensive Disorders. *JAMA Netw Open*. Published June 13, 2024.
doi:10.1001/jamanetworkopen.2024.16844

Data
Data available: Yes
Data types: Deidentified participant data
How to access data: farah.h.amro@uth.tmc.edu
When available: With publication

Supporting Documents
Document types: None

Additional Information
Who can access the data: researchers
Types of analyses: N/A
Mechanisms of data availability: N/A
